# COP26: Looking forward from Glasgow by placing health at the center of climate action

**DOI:** 10.1016/j.joclim.2022.100117

**Published:** 2022-02

**Authors:** Marcalee Alexander, Mohamed Eissa, Ruth McDermott-Levy, Rhiannon Osborne, Elizabeth Pleuss, Poornima Prabhakaran, Cecilia Sorensen

**Affiliations:** a Sustain Our Abilities, United States; b Department of Physical Medicine and Rehabilitation, University of Alabama at Birmingham School of Medicine, United States; c Department of Physical Medicine and Rehabilitation, Harvard School of Medicine, United States; d Liaison Officer for Public Health Issues, International Federation of Medical Students’ Associations, Denmark; e M. Louise Fitzpatrick College of Nursing, Villanova University, United States; f School of Clinical Medicine, University of Cambridge, United Kingdom; g Students for Global Health, United Kingdom; h Global Health Bureau, USAID, United States; i Head-Environmental Health and Additional Professor; Deputy Director, Centre for Environmental Health, Public Health Foundation of India, India; j Senior Research Scientist, Centre for Chronic Disease Control, India; k Global Consortium on Climate and Health Education, Columbia University, United States; l Department of Environmental Health Sciences, Mailman School of Public Health, United States; m Department of Emergency Medicine, Columbia University Irving Medical Center, United States

Climate change is both the greatest threat and health opportunity humanity will face in the foreseeable future. At the recently concluded Glasgow Climate Summit-COP26, the connection between health and climate change and the need for concerted action was galvanized, by no small feat. In the months prior to COP26, an unprecedented momentum within the health sector was mounted to provide a substantive impetus for health workers and institutions to support calls for drastic climate action. Initiatives such as the healthy climate prescription letter [1], signed by organizations from 91 countries representing 46 million doctors, nurses and other health care professionals was a fervent “Call to Action'' to motivate governments to take urgent steps to address the climate crisis. Additionally, work by The Global Climate and Health Alliance, which graded and ranked individual countries on the inclusion of health in their nationally determined contributions (NDCs), stimulated a deeper discussion around the importance of “Healthy NDCs” and created a platform for advocacy. At COP26, momentum accelerated through a health pavilion hosted by the World Health Organization (WHO), which emphasized the importance of the intersection between health and climate. A powerful and significant step forward was made when over fifty countries around the world, including the United States, made commitments aligned with the COP 26 Health Program and to follow the UK National Health Service to transition to climate resilient and low-carbon health systems [2]. However, despite this mobilization, COP26 did not deliver sufficient climate action. Existing pledges, if fulfilled, will result in well over 2° of global warming, and key issues such as loss and damage for developing countries were sidelined.

Health has now officially entered the interchange at the COP and the message of the health community is urgent and clear – health and equity must be fundamentally integrated into climate policy within all sectors and national and international commitments are needed to ensure our health systems are resilient and responsive in a changing climate. During the Global Climate Change and Health Conference, a side-event hosted by the WHO, health delegates deliberated with representatives and domain experts from governments, industries, and vulnerable constituencies about the role of health with respect to energy, transport, food, nature, and finance and how health needs could be more closely incorporated with sectoral policies. Similarly, an initiative facilitated by the Health and Climate Network enabled inter-sectoral communication regarding climate resilient health systems. We provide an overview of these dialogues and outcomes as salient discussion points to assist the international health community to contribute to the global climate discourse.

## Energy

Detrimental impacts from the combustion of fossil fuels are immense. The extraction of fossil fuels has, for decades, resulted in violence, pollution, and displacement at the sites of natural resources. High disease symptomatology and emotional distress has been documented in communities near oil extraction sites in the Niger Delta [3]. Moreover, the murder and violence faced by women land defenders protecting land from extractive industries was highlighted at a vigil at COP26. Exposure to air pollution is the 4th leading cause of death worldwide [4] and researchers have estimated that air pollution caused by the extraction and burning of fossil fuels annually kills more than 8 million people globally [5].

Fossil fuels are the leading cause of climate change [6]. Despite this, and the work of a new coalition led by Denmark and Costa Rica (the ‘Beyond Oil and Gas Alliance’), the final text of the Glasgow Climate Pact fails to clearly name fossil fuels as the main culprit driving climate and fails to call for the complete phase out of their use. Instead of an unambivalent call for elimination of fossil fuels, the final COP26 recommendations call for “efforts towards the phasedown of unabated coal power and phase-out of inefficient fossil fuel subsidies” [7]. While the explicit reference to fossil fuels is the first to appear in a final COP document, this weak language represents a missed opportunity to improve human health by reducing air pollution, the occupational risks of the extraction industry, and the overall health consequences of climate change.

The co-benefits to health of a just transition to renewable energy, such as decreased morbidity and mortality, the creation of high-quality jobs, and increased energy accessibility are manifold [8], but the power of the fossil fuel industry is an ever-present barrier to realizing these benefits. While tobacco companies have long been excluded from WHO negotiations, fossil fuel lobbyists represented the largest delegation to COP26 [9]. Similar to the mobilization of the health voice regarding the harms of tobacco use, the healthcare community must unite globally and demand the urgent prioritization of people's health over profits that accrue to the fossil fuel industry and emphasize the monumental, short- and long-term physical, mental health and economic benefits humanity will gain by rapid transition to a low-carbon energy sector.

## Transport

Transportation contributes 15–20% of greenhouse gas emissions globally. Gasoline and diesel fueled vehicles pollute the air and over-reliance on the automobile for local transportation limits opportunities for active transportation such as walking, biking, and wheeling. These risk factors – air pollution and physical inactivity – are linked to leading causes of mortality globally: ischemic heart disease, respiratory disease, and diabetes. Creation of accessible safe active transport routes and public transportation will support a reduction in green-house gases (GHGs) and health co-benefits. The health community can leverage this information and support a transition to more carbon-neutral transportation through advocacy and collaboration with community planners and transportation specialists ensuring transportation is accessible, safe and affordable to all. Furthermore, in our own practices, we have the opportunity to re-envision low-carbon and low-transport health care delivery, via the use of telemedicine which can dramatically reduce carbon outputs from travel [10].

## Food systems and nature

Food systems are responsible for approximately one third of global emissions [11]. Industrial agriculture is one of the largest global causes of deforestation, a key driver of climate change [12]. The use of large swaths of land for industrial farming has eroded forests globally which serve as carbon sinks, support unique ecosystems, serve as sources of local cooling, and support mental and physical health of local communities [13]. Industrial agriculture and food production is responsible for the significant loss of livelihoods, land rights and health of indigenous communities across the world, who despite being only 5% of the global population steward 80% of the world's biodiversity. Loss of forests and land from intensive industrial farming practices and food systems also increase the risk of pandemic potential diseases emerging by removing natural barriers to the transfer of zoonotic infection [14].

Excess consumption of meat and dairy is not only detrimental to the environment but also detrimental to health, leading to increased cardiovascular diseases, cancer, and diabetes. Communication regarding the health benefits of plant-based diets is an opportunity for health care professionals not only to help improve the health of populations but also to decrease the generation of fossil fuels and deforestation that occur from the excess consumption of red meats. Similarly, communication and promotion of the benefits of outdoor, nature-based activities as a means of medical and psychological therapies also can lead to lower carbon emissions and a healthier population [15].

## Financing

International financing to address the climate crisis falls far short of what is needed. The commitment made twelve years ago by high income countries to mobilize $100 billion a year for mitigation and adaptation efforts by developing countries has not been fulfilled, yet this funding is desperately needed for countries anticipated to experience the worst impacts to adapt and transition to a low carbon pathway. High-income and high GHG emitting countries must do their part to transfer financing. Regrettably, while these promised funds for climate action have failed to materialize, over 180 billion dollars has been spent on fossil fuel subsidies annually [16] and since the Paris Agreement, banks have invested more than 3.6 trillion dollars into fossil fuels [17].

The recommitment to deliver the $100 billion annually and to end fossil fuel subsidies through COP 26 is welcome. As the post 2025 financing commitment is developed, health advocates should join the call for increased financing for adaptation, especially adaptations that protect human health from climate change impacts. Financial provision based upon historical contributions to the climate crisis [18] must also be made to compensate nations already faced with loss and damage, including health impacts. A financing facility for loss and damage was proposed at COP26 but tabled until COP27. Mechanisms to overcome barriers to technology transfer of green innovations and healthcare to less-developed countries, including intellectual property barriers, trade rules, and licensing are also sorely needed.

Financing for research and education related to climate change and health must be increased. To ensure effective and appropriate changes are made to our healthcare systems and the environments of the populations we serve, we need systematic research about the impacts of climate change on health in specific locales and among specific populations (including vulnerable sub-populations, individuals with mental illness or sensory dysfunction, the elderly, children, pregnant people, people with limited mobility, and those with chronic illnesses).The Research Gaps and Priorities paper produced by WHO in collaboration with members of the WHO Civil Society Working Group to Advance Action on Climate and Health released at COP 26 highlighted the inequitable distribution of global climate change and health research with the greater part of research focused on high-income countries and a less than requisite focus on more vulnerable countries and populations [19]. Forging partnerships that facilitate and promote both funding and capacity-building for research on climate change and health where it is most needed is an important next step.

### Strategies going forward

As we collectively reflect on the health agenda at the COP 26 conference and strategize for the future, it is imperative the health community accelerates organization and mobilization for climate action. In addition, we must engage and decarbonize our own health systems. Fifty countries have pledged to transition to climate resilient and low-carbon health systems. We must hold our policy makers accountable where commitment has been made and advocate for a similar pledge within countries that have not yet joined. Building low-carbon, climate resilient health systems must be a key consideration of post-pandemic economic recovery. Additionally, we must work within our own institutions to build a climate-ready health workforce, by integrating climate and health into health professional training programs for pre- and post-licensure practitioners. Ultimately, we must transition to a system where healthcare practitioners include prevention and treatment of climate related illness in their daily work. This must go hand in hand with strengthening our capacities to mount a suitable adaptation response to the health impacts of continuing climate events. In addition, it is important to reflect on who is considered part of the health community. Traditional healers, land defenders, frontline communities, community organizers and individuals living with chronic health concerns are all essential voices in the fight for climate and health, and their perspectives must be better represented within the health community. Most importantly, we must advocate the fact that climate and health justice is a right for everyone, rather than a privilege. To further this agenda and the vision for “health in all sectors,” communication and collaboration within all aspects of the health community is imperative.

We must set concrete plans for the inclusion of health in climate action. As part of health advocacy at COP26, tens of country delegations were approached regarding the inclusion of health in their climate action plans and the final text of the Glasgow Climate Pact; however, many decision makers and negotiators were not ready to listen about health. The designation of health as a theme of the COP26 Presidency Program, the presence of a health constituency and tangible policies and plans for negotiations on health and climate change may have facilitated communication. Moving forward it is crucial that international health and climate change communities consistently communicate and collaborate with each other to set tangible and concrete outcomes that need to be achieved at the COP and that can be presented and used by negotiators on the decision-making table. Still these actions must not be solely focused on COP. Concomitantly, communication and education about the linkage between climate change and health to the general population and community leaders must be greatly expanded by health care professionals at local, regional, state, national and specialty-based levels. Moreover, health professionals must advocate suppliers in the health care industry make similar commitments regarding the need for education.

In addition to improving communication about climate change and health, it is crucial to bridge gaps between fields by creating intersectoral collaborations and workstreams focused on shared visions. Compared to other groups, healthcare constituents are late to the climate agenda, engagement in COPs, climate advocacy and coordinated actions. Most important climate decisions occur outside of international conferences, and the health community must learn to act in conjunction with like-minded groups holding national governments to account. It is important health providers develop relationships with other sectors of the climate change community and obtain a working understanding of their knowledge and priorities. Simultaneously, as we mold shared agendas, we must focus our efforts on engaging with colleagues in other sectors and responding to their needs and concerns regarding planning and development of strategic approaches, which may vary greatly between countries and locales. This shared crisis needs shared responsibility which can only be achieved in an intersectoral and interprofessional approach.

On an ongoing basis, it is paramount that the healthcare community strategize together to help solve the climate crisis. Considering the urgency of the crisis, The Journal of Climate Change and Health will publish two special issues in 2022: one on optimizing communication and one on optimizing collaboration with regards to climate change and health. As we move forward, it is imperative we communicate and engage year-round with the communities most affected by climate change. The healthcare community must foster inclusivity and give voice and agency to vulnerable groups along with the general population, working in tandem with and representing the needs of all people. As the health profession, we have a duty to engage in advocacy, research and policy that aims to mitigate and adapt to the climate crisis in a just and fair way, recognizing those least responsible for the crisis are the most vulnerable to its impacts.

Still, we must go further – we must dissect the systems of inequality, consumption and power that have been damaging health for decades and have resulted in the climate crisis. This is imperative to help forge a healthier worldwide civilization. The climate crisis is a symptom of our world, one that values profits over the health of its inhabitants and our planet, and, as the health community, we must move forward in solidarity to create a more equitable and lasting future for all.

## Declaration of Competing Interest

The authors declare that they have no known competing financial interests or personal relationships that could have appeared to influence the work reported in this paper.
